# Bronchus to Caecum—An Unusual Case of a Migratory Aspirated Dental Implant

**DOI:** 10.1002/rcr2.70431

**Published:** 2026-01-05

**Authors:** Frank Yong, Hanson Siu, Philip G. Bardin

**Affiliations:** ^1^ Monash Lung Sleep Allergy & Immunology, Monash Health Clayton Victoria Australia

**Keywords:** aspiration, bronchoscopy, colonoscopy, foreign body, migration

## Abstract

Foreign body aspirations more commonly occur in children and the elderly. There are many challenges in their diagnosis and management, with both acute and chronic complications. Bronchoscopy is the preferred management approach for airway evaluation and retrieval of an aspirated foreign body. The availability of ultra‐thin bronchoscopes, advanced imaging, and robotics allows interesting new options for the management of foreign body aspiration. An ingested foreign body tends to pass naturally through the gastrointestinal tract but may also require endoscopic removal in case of complications. We explore the challenges in the management of an aspirated dental implant that migrated from the bronchial tree to the gastrointestinal tract in this unique case.

## Graphical abstract

We present a unique case of an 83‐year‐old man who aspirated an endosteal implant during a dental procedure. The foreign body was initially in the bronchus but later migrated to the gastrointestinal tract. This case explores the challenges of clinical management and retrieval of foreign bodies in the respiratory and gastrointestinal tract.

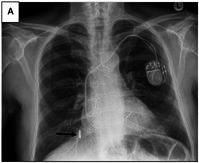

## 1 Introduction

Foreign body aspiration tends to occur in children and the elderly rather than healthy adults [1]. Most adult patients with foreign body aspiration have identifiable risk factors, including neurological deficits with swallowing difficulties, neuromuscular disease, altered mental status, or intoxication [2]. These foreign bodies may be composed of metallic or plastic materials, but most commonly are organic matter such as bone or vegetables [2]. Dental procedures are the most common source of iatrogenic foreign body aspirations because they require local anaesthesia with loss of upper airway perception and decreases in swallow efficacy [2]. The patient's age, presence of saliva, and difficulty in direct visualisation increase the risk of aspiration. Aspirated items during dental procedures include teeth, surgical drills, implants, and instruments [3].

Clinical presentation of foreign body aspiration can range from acute airway obstruction requiring urgent intervention to indolent cough. There is not always a definite choking episode. Most patients report chronic symptoms including cough, dyspnoea, fever, and haemoptysis [4]. If the sentinel event is overlooked, these respiratory symptoms can mimic other diseases such as asthma, chronic obstructive pulmonary disease, and heart failure [4]. Retained foreign bodies can cause recurrent pneumonias, lung abscesses, bronchiectasis, and other complications [1]. Timely extraction of aspirated foreign bodies is crucial to alleviate acute symptoms and prevent long‐term complications.

Rigid or flexible bronchoscopy is the preferred management approach for airway evaluation and retrieval of the aspirated foreign body depending on its location and its properties [1, 2]. However, with ongoing development in the field of interventional bronchoscopy with the increasing availability of ultra‐thin bronchoscopes, advanced imaging, and robotics, the options for management of foreign body aspiration broaden. Peristalsis tends to pass any ingested foreign body naturally through the gastrointestinal tract, but complications may arise that warrant endoscopic retrieval [5]. In this unique case, we will explore the challenges in managing an aspirated dental implant that migrated from the bronchial tree to the gastrointestinal tract.

## 2 Case Report

An 83‐year‐old gentleman was referred for assessment following possible aspiration of an endosteal implant during a dental procedure. Past medical history was significant for coronary artery bypass graft, permanent pacemaker, type 2 diabetes mellitus and peripheral vascular disease. The patient reported intermittent cough but had no wheeze, haemoptysis or dyspnoea. There were no pre‐existing issues with swallowing and no history of aspiration. Vital signs were unremarkable. Physical examination found no evidence of stridor with good bilateral air entry on auscultation.

**FIGURE 1:**
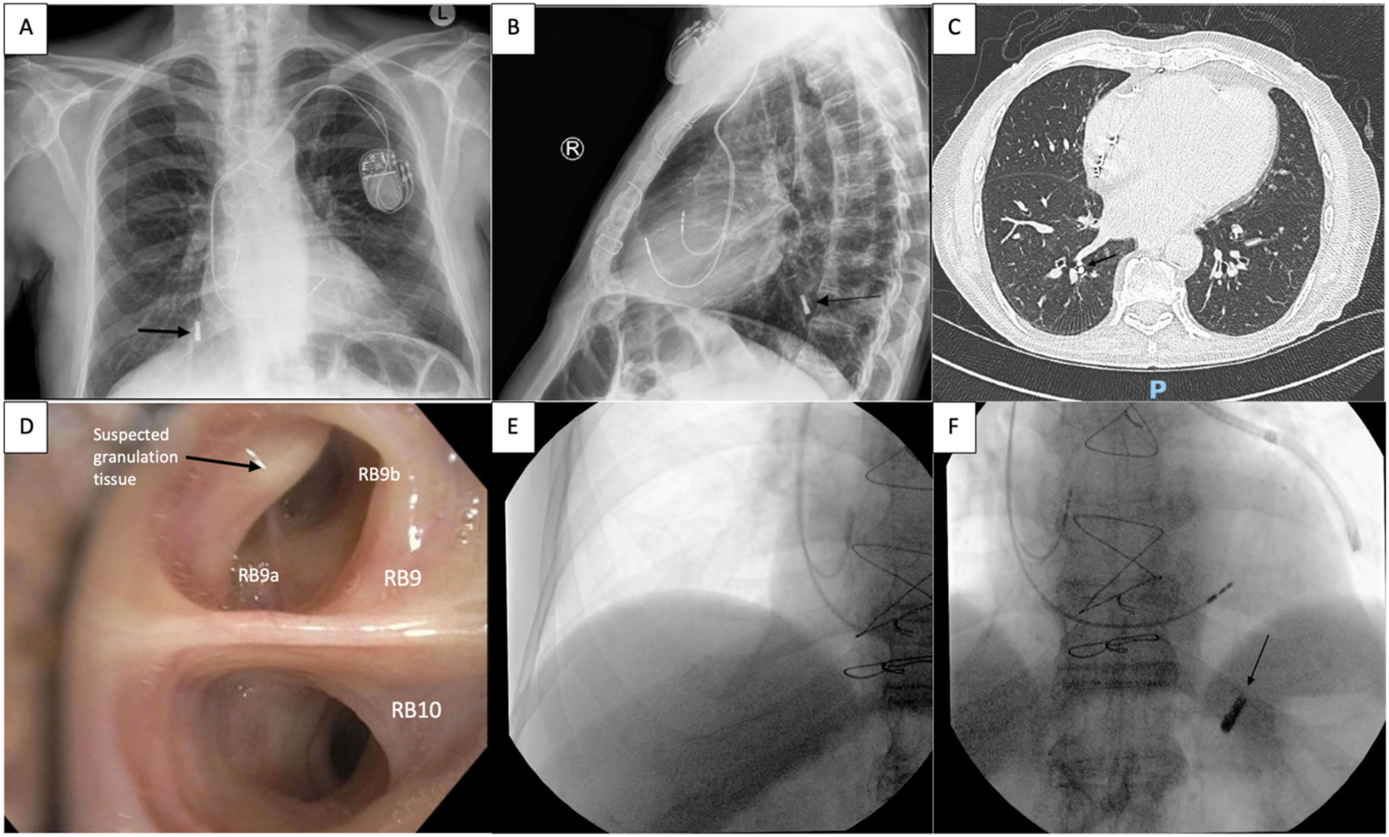
Respiratory phase: (A, B) Chest X‐ray showing metallic foreign body in the right lower lobe → (C) CT Chest confirming foreign body within distal posterior basal segment of right lower lobe → (D) Bronchoscopic view of right lower lobe showing suspected granulation tissue in RB9 and failure to identify foreign body → (E, F) Fluoroscopic migration of foreign body to left hemithorax.

Chest X‐ray (Figure 1A,B) revealed a 15 × 5 mm metallic foreign body in the right lower lobe. Computed Tomography (CT) of the chest (Figure 1C) confirmed the dental implant lying within the distal posterior basal segment bronchus of the right lower lobe (within RB10) associated with minor distal mucous plugging. In consultation with cardiothoracic surgery, the foreign body was deemed too distal to be confidently retrieved via rigid bronchoscopy. Elective bronchoscopy was organised; however, given the patient's minimal symptoms and insistence that he could ‘cough it out’, the patient was reluctant to be admitted for observation and opted for discharge despite the risks.

Interrogation of the bronchial tree proceeded with a standard Olympus flexible bronchoscope (distal outer diameter 4.8 mm and working channel width 2.0 mm). At our facility, there was no access to ultra‐thin bronchoscopes or robotic bronchoscopy to visualise the distal subsegmental airways. The foreign body was not identifiable in the right lower lobe with suspected granulation tissue at the entrance of RB9 (Figure 1D). Further exploration of the rest of the bronchial tree failed to find the dental implant. Given the suspicion that the foreign body was lodged deeper in RB10 subsegments on CT, intra‐procedural fluoroscopy was employed to pinpoint its location. Interestingly, there was no sign of the dental implant in the right hemithorax (Figure 1E). Rather, the dental implant was now in the left hemithorax (Figure 1F). It was difficult to ascertain whether it was in the distal segments of the left lower lobe or had migrated into the abdomen—the patient was admitted for further imaging and observation. Further referral to cardiothoracics or an alternate centre with ultra‐thin bronchoscope was considered.

**FIGURE 2:**
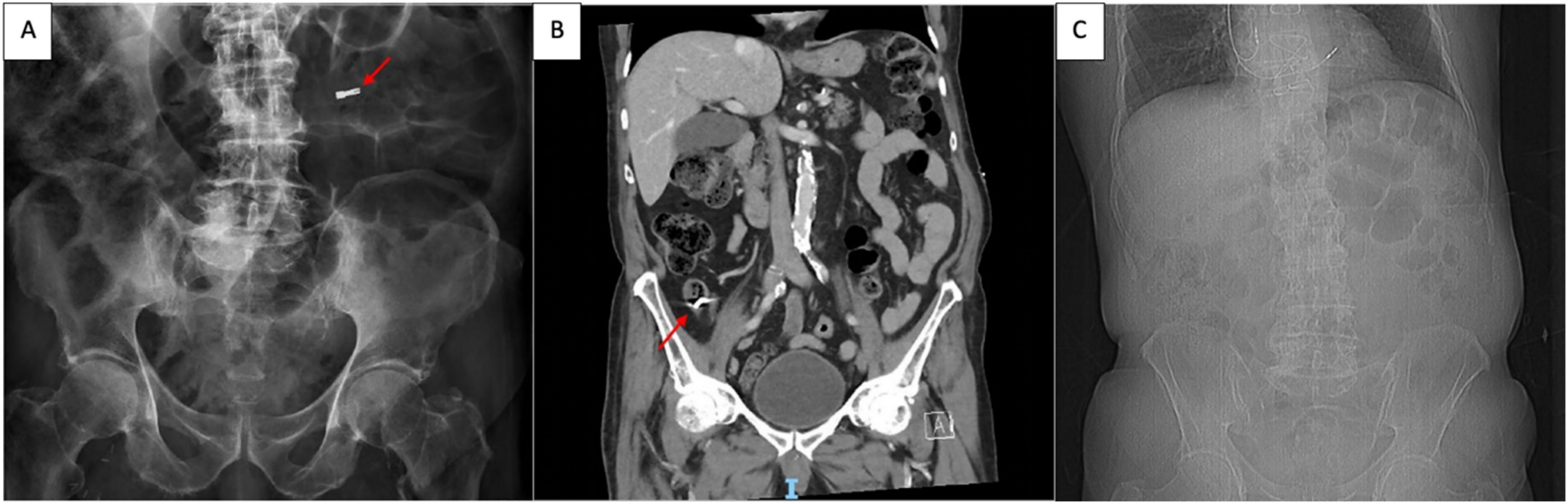
Abdominal phase: (A) Abdominal XR showing metallic foreign body in the abdomen → (B) CT abdomen showing impaction at ileo‐caecal valve → (C) CT scout confirming spontaneous passage of foreign body.

To our surprise, a subsequent abdominal radiograph confirmed that the foreign body was now in the left abdomen (Figure 2A). In consultation with gastroenterology, serial imaging was preferred to endoscopic evaluation due to the likelihood of it passing naturally. Outpatient abdominal radiographs showed that the foreign body had not passed for 2 months, and the patient was commenced on laxatives by his primary care physician. Unfortunately, the patient was re‐admitted due to worsening abdominal pain, with the dental implant impacted at the ileo‐caecal junction CT of the abdomen (Figure 2B). Endoscopic evaluation by colonoscopy failed to visualise and retrieve the object due to faecal material despite adequate bowel preparation. Follow‐up abdominal imaging was organised and a plan for repeat colonoscopy after comprehensive bowel preparation was organised. Subsequent imaging demonstrated that the dental implant was no longer present and had successfully passed without further endoscopic intervention.

## 3 Discussion

There have been two previous case reports that describe a similar finding of foreign body migration from the respiratory to the gastrointestinal tract [6, 7]. We postulate the most likely mechanism to be cough‐induced swallowing of the foreign body after dislodgement from the bronchial tree based on the clinical picture and sequence of events. An alternative but less likely explanation could be direct passage from the pleura and diaphragm into the abdomen. There were no signs of peritonism, and the blunt nature of the dental implant makes this highly unlikely. Previous reports involving a pushpin and sewing needle offer similar thoughts.

Our patient presented early given the suspicion of iatrogenic aspiration of a dental implant during the procedure. This allowed for prompt radiographic confirmation of the metallic radiopaque object on chest X‐ray (Figure 1A,B). Organic and plastic objects are more difficult to identify, and changes may also be obscured by chronic parenchymal change. Radiographic features on computed tomography may also show focal bronchiectasis, atelectasis, non‐resolving pneumonia, and unilateral hyperinflation [8].

Bronchoscopy is the preferred management approach for airway evaluation and retrieval of aspirated foreign bodies. Flexible conventional bronchoscopy is generally used as the initial procedure as it allows for comprehensive airway evaluation and has an overall 90% success rate for retrieval [1, 2]. Compared with rigid bronchoscopy, it is also more accessible, less expensive, and general anaesthetic is not required. Further, preservation of the cough reflex is potentially helpful in the removal of a foreign body. Rigid bronchoscopy offers superior airway control, suction and extraction capabilities, and is used more commonly in children [9].

The foreign body was found to be within the subsegments of the posterior basal segment of the right lower lobe, which was deemed too distal and small for rigid bronchoscopy. Our facility did not have access to ultra‐thin bronchoscopy, so we proceeded to interrogate the bronchial tree with a standard flexible bronchoscope (4.8 mm diameter and 2.0 mm working channel width) in hopes that the dental implant may have dislodged more proximally. Interestingly, there was suspected granulation tissue found at the entrance of RB9 rather than RB10, which could support our suspicion that the implant had migrated. Alternatively, we could have referred the patient to other centres with more procedural expertise and equipment access, or to cardiothoracic surgery for surgical removal, had the foreign body remained lodged in the distal right lower lobe after initial standard bronchoscopy.

The initial location of the foreign body could have been accessible with a trained proceduralist adept at navigating the subsegmental airways with an ultra‐thin bronchoscope. Ultra‐thin bronchoscopes have an outer diameter ≤ 3.0 mm and are equipped with a working channel width ≤ 1.7 mm [10] and are increasingly used to access and sample peripheral lung lesions with high diagnostic value [10]. However, their use in the management of foreign bodies has been rarely reported. Yuan et al. [11] describe the intense manual navigational planning required to locate an aspirated chilli in LB10ciiβ and the difficulty in using mini forceps in such a small space. They employed a 1.1 mm ultrathin cryoprobe to successfully remove the organic foreign body. This highlights the complexity and challenges that arise with more distally lodged foreign bodies and the potential utility of ultra‐thin bronchoscopes.

In addition to the diameter of the bronchoscope, various endoscopic instruments can be utilised during bronchoscopy to facilitate the removal of the foreign body depending on its size, structure, and properties. This includes forceps, baskets, snares, cryoprobes, and magnetic probes. Whilst cryoprobes are effective for organic objects with significant surrounding granulation tissue, magnetic probes can assist in retrieving ferromagnetic objects in the distal airway [9]. This may have been useful in our case of a metallic dental implant. However, the patient had a permanent pacemaker in situ that precluded this option.

We found that the foreign body had migrated from the subsegments of RB10 to the left hemithorax with intra‐procedural fluoroscopy at the time of bronchoscopy. Intra‐procedural fluoroscopy is normally used to assist in accessing peripheral lung nodules [12] and may be beneficial if the aspirated foreign body cannot be located endoscopically. In our case, this was useful in confirming that the foreign body was no longer in the right hemithorax to guide intra‐procedural management. Advances in navigational imaging and robotics could also change the way we approach distal foreign objects in the future.

Ingested foreign bodies are a common problem and usually require limited intervention. Generally, 80%–90% of ingested foreign bodies successfully pass without intervention. Approximately 10%–20% must be removed endoscopically, and ~1% require surgery [5]. The clinical management of foreign body ingestion depends on the size, sharpness, location, and properties of the object. During the gastrointestinal phase of our case, serial imaging was preferred to immediate endoscopic interrogation due to the likelihood of the foreign body passing with laxatives. The foreign body was also blunt in nature and radiopaque, allowing for simple radiography monitoring. The patient was also elderly and opted for less invasive measures.

However, ingested foreign bodies may be obstructed at potential sites of anatomic narrowing, including the oesophageal sphincters, pylorus, and ileocaecal valve [13]. Retained foreign body complications in the gastrointestinal system include perforation, obstruction, infection, and fistula formation [14]. Our patient's dental implant remained impacted at the ileo‐caecal valve with representation that prompted further endoscopic evaluation. Endoscopic removal failed to visualise or remove the foreign body due to abundant faecal material despite adequate bowel preparation, highlighting the challenges that arise with ingested foreign bodies. Fortunately, our patient's dental implant eventually passed spontaneously from the gastrointestinal system—a journey of more than 2 months after initial aspiration.

In conclusion, our case details an unusual series of events following an aspirated dental implant that migrated from the bronchial tree to the alimentary tract by probable cough‐induced dislodgement. Beyond an extremely rare occurrence, this case highlights the challenges in clinical management of foreign bodies in the respiratory and gastrointestinal tracts.

## Author Contributions

**Frank Yong:** writing, editing, and final preparation of manuscript. **Hanson Siu:** review, editing, and final preparation of manuscript. **Philip G. Bardin:** conception and design of the case report and final review of the manuscript.

## Funding

This work was supported by Monash Lung and Sleep Institute.

## Consent

The authors declare that written informed consent was obtained for the publication of this manuscript and accompanying images using the form provided by the Journal.

## Conflicts of Interest

The authors declare no conflicts of interest.

## Data Availability

Data sharing not applicable to this article as no datasets were generated or analysed during the current study.
